# Migration to middle-income countries and tuberculosis—global policies for global economies

**DOI:** 10.1186/s12992-017-0236-6

**Published:** 2017-03-15

**Authors:** Julia Moreira Pescarini, Laura Cunha Rodrigues, M. Gabriela M. Gomes, Eliseu Alves Waldman

**Affiliations:** 1 Faculdade de Saúde Pública, Universidade de São Paulo, Av. Dr. Arnaldo, 715, São Paulo, SP 01246-904 Brazil; 2 Department of Infectious Disease Epidemiology, Faculty of Epidemiology and Public Health, London School of Hygiene and Tropical Medicine, Keppel St, London, WC1E 7HT UK; 3 Liverpool School of Tropical Medicine, Pembroke Place, Liverpool, L3 5QA UK; 4 CIBIO-InBIO, Centro de Investigacao em Biodiversidade e Recursos Geneticos, Universidade do Porto, Rua Padre Armando Quintas, n° 7, Vairão, 4485-661 Portugal; 5 Instituto de Matematica e Estatistica, Universidade de São Paulo, R. do Matão, 1010 - Vila Universitaria, São Paulo, SP 05508-090 Brazil

**Keywords:** Tuberculosis, Migration, Health equity, Low- and middle-income countries

## Abstract

**Background:**

International migration to middle-income countries is increasing and its health consequences, in particular increasing transmission rates of tuberculosis (TB), deserve consideration. Migration and TB are a matter of concern in high-income countries and targeted screening of migrants for active and latent TB infection is a main strategy to manage risk and minimize transmission. In this paper, we discuss some aspects of TB control and migration in the context of middle-income countries, together with the prospect of responding with equitable and comprehensive policies.

**Main body:**

TB rates in middle-income countries remain disproportionally high among the poorest and most vulnerable groups in large cities where most migrant populations are concentrated. Policies that tackle migrant TB in high-income countries may be inadequate for middle-income countries because of their different socio-economic and cultural scenarios. Strategies to control TB in these settings must take into account the characteristics of middle-income countries and the complexity of TB as a disease of poverty. Intersectoral policies of social protection such as cash-transfer programs help reducing poverty and improving health in vulnerable populations. We address the development of new approaches to improve well-established strategies including contact tracing and active and latent TB screening as an ‘add on’ to the existing health care guidelines of conditional cash transfer programs. In addition, we discuss how it might improve health and welfare among both poor migrants and locally-born populations. Authorities from middle-income countries should recognise that migrants are a vulnerable social group and promote cooperation efforts between sending and receiving countries for mitigation of poverty and prevention of disease in this group.

**Conclusions:**

Middle-income countries have long sent migrants overseas. However, the influx of large migrant populations into their societies is relatively new and a growing phenomenon and it is time to set comprehensive goals to improve health among these communities. Conditional cash transfer policies with TB screening and strengthening of DOTS are some strategies that deserve attention. Reduction of social and health inequality among migrants should be incorporated into concerted actions to meet TB control targets.

## Background

Tuberculosis (TB) rates have declined significantly in the 20th century worldwide, but HIV and multidrug-resistant tuberculosis (MDR-TB) have had drastic effects in some of the poorest countries [1, 2]. TB burden is still much higher in the poorest economies and highly concentrated among vulnerable^1^ populations and specific groups such as homeless people, people living with HIV (PLHIV), prisoners and migrants [1, 3]. It has been a century of reawakening concerns about TB control and rebuilding strategies for the next decades.

**Fig. 1:**
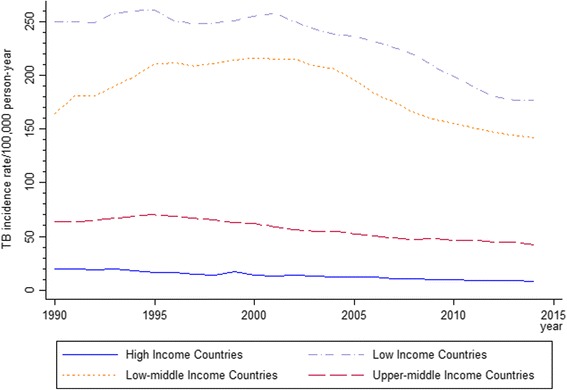
Median TB incidence rate/100,000 person-year for each group of country (High, Upper-middle, Low-middle and Low-Income Countries)^*^ 1990–2014. ^*^Median incidence was calculated using panel data, collapsing countries and calculating the median TB incidence by group of country/year. Source: Tuberculosis data extracted from WHO [1]. World Bank [4] was used to classify country groups

TB and TB/HIV coinfection partially reflects the income and development level of a country, with medium incidence rates at least 20 times higher in low-income countries (LIC) than in high-income countries (HIC) (Fig. 1) [1, 4]. Incidence of TB and associated mortality are now low in most HIC, with TB risk being disproportionally high among migrants from high burden countries—up to 80% of cases reported in some receiving countries [5]. Migrants are a risk group for TB from the perspective of the receiving country as TB incidence rates among them usually reflect TB incidence in the country of origin [6].

In HIC, migrants from high TB burden countries typically maintain higher active TB prevalences compared to the local population and, TB prevalence can reach at 3.3/1000 among migrants [7]. It may reflect a higher proportion of latent TB infection (LTBI) and increased risk of disease reactivation potentially increasing the risk of transmission to local communities. Migrants are often a hard-to-reach group, and thus poses a major challenge for TB control in high-income countries.

Over the last 15 years, the economic development of Upper-middle income countries (UMIC), especially the BRICS (Brazil, Russia^2^, India, China and South Africa) [1], has led to an increase in health spending and strengthening of primary care services [8]. Many regions have either achieved or made gains in achieving the TB-related Millennium Development Goals [1], but in contrast to the developed world, TB rates in some countries have remained high due to sustained poverty and poor living conditions [9]. Today, a great difference in TB rates remains between UMIC and Low-middle Income countries (LMIC) (Fig. 1).

Most South American countries have robust TB control programs, but inner-city populations often live in areas of deprivation with high unemployment rates and high rates of TB-related morbidity and mortality [1]. This is common even in countries with sound economic development such as Argentina, Brazil and Chile that attract a high number of regional migrants from other middle-income countries [10–12]. Migration and TB control strategies in middle-income countries, especially in UMIC, need further investigation taking into consideration this context.

## Migration, vulnerability and TB

**Fig. 2:**
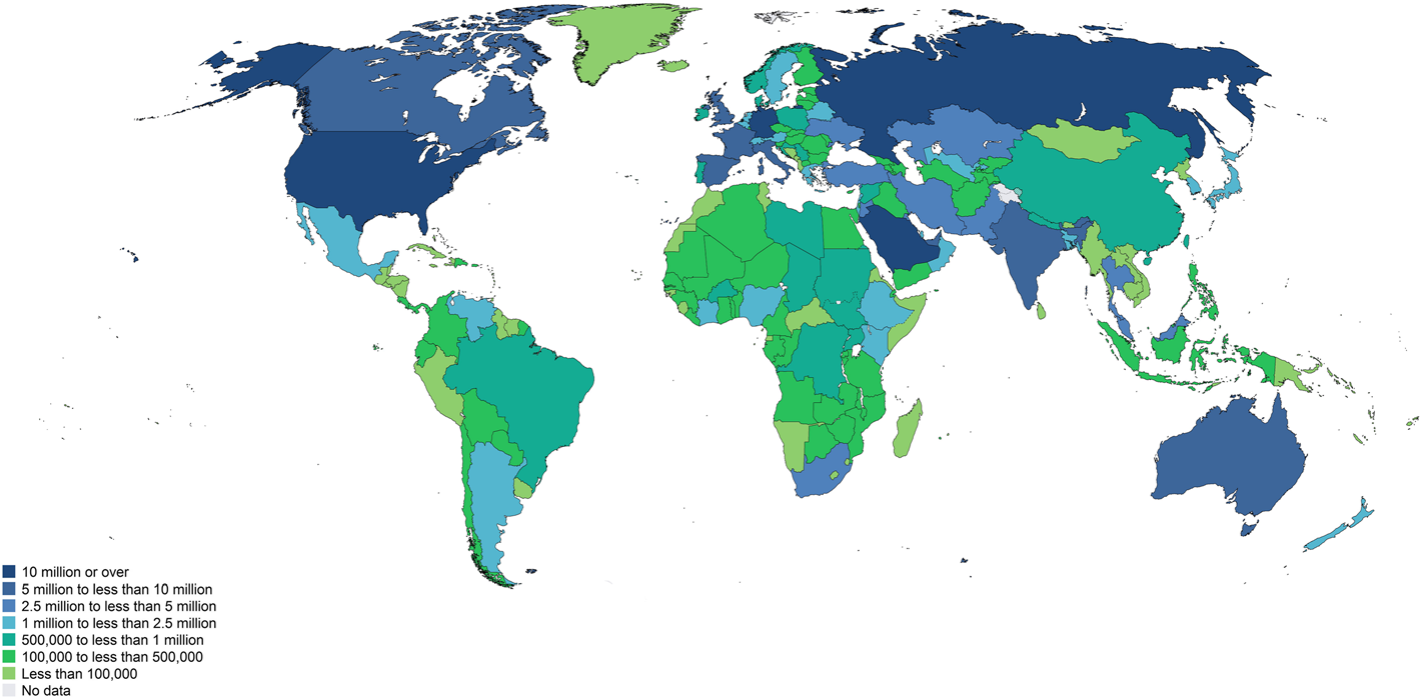
Number of international migrants in 2015. Source: Adapted from United Nations (UN, [13])

As part of economic globalization, since the 1980s there has been an intensification of migration. Migrants living in developing regions accounted for 42% of total migration stock in 2015 and South-South^3^ migration now comprises 36% of total migration, a proportion that is higher than that of South–north migration [13]. Figure 2 shows total number of migrants by country according to UN [13]. Refugees from conflict areas and natural disasters are highly visible in the media, but half of the migration stock (106 million) are international labour migrants who may live in an irregular situation in the receiving country [14]. Violence, labour exploitation and sexual harassment are frequently reported as occurring during the migration journey and in the country of destination [2, 14]. Stressful conditions and social vulnerability can contribute to the development of non-infectious and infectious diseases in migrants, including TB infection or LTBI reactivation [15, 16].

Host societies are increasingly concerned with social and economic impacts of migration including potential effects on morbidity and mortality and the burden on health care services (especially when accurate migration data are not available) [14]. The recent international financial crisis and austerity policies in the developed world have accentuated social inequities and affected migrants disproportionately [15].

Migration in middle-income countries is generally directed to major cities [17, 18]. Migrants are more likely to settle in low-income inner-city neighbourhoods with the greatest TB burden and some migrant groups often face more poverty, vulnerability and social exclusion than native communities living in the same areas [19]. In large cities in middle-income countries with large inequalities and high proportions of HIV, homeless individuals and drug users [1, 5], migrants comprise a new risk group for TB burden that needs to be specifically targeted.

## Tuberculosis control among migrants

### Screening for active and latent TB infection

In addition to strengthening Direct Observed Treatment, Short-Course (DOTS) strategy and Bacillus Calmette–Guérin (BCG) vaccination among migrants since the 1980s, many high-income countries with low TB burden have also incorporated active and latent TB screening. TB screening strategies directed to migrants are aimed to reduce TB burden and the risk of TB transmission: migrant screening for LTBI is estimated to prevent many new TB cases [20], potentially reducing the risk of transmission in the community whereas screening for active TB is thought to prevent more severe forms of TB and reduce treatment costs [21]. There is no consensus on the most appropriate screening strategy: screening for active TB and/or LTBI; pre-arrival, post-arrival or screening in the community; use of chest X-rays (CXR) and/or IGRA [5]. Screening strategies based on TB incidence in the country of origin or on the type of migration—asylum seekers/refugees—is also a common approach [22]. The cost-effectiveness of each screening strategy is determined by TB epidemiology in the country of origin or type of migration [16].

While some HIC have chosen compulsory screening for some types of migrants, others do not consider screening [22]. The decision for not performing screening is usually motivated by lack of financial aid, low yield in areas with high presence of irregular migrants (who are less frequently screened), and ethical considerations [22, 23].

The strategies for TB control in migrant populations in middle-income countries are poorly discussed in the literature. TB control in middle-income countries is more complex due to multifaceted problems such as poverty, inequality and higher infectious disease rates in locally born populations. It is known that the prevalence of LTBI is likely to be higher in low- and middle-income countries than in high-income countries. In Brazil, active and latent TB screening is only suggested for contacts of TB patients, for HIV-infected individuals, the homeless and prisoners (and treatment for LTBI is not always recommended) [24]. The estimated prevalence of LTBI was 60% among contacts of TB patients living in urban areas [25]. To consider migrants from high-incidence TB countries as risk groups for TB and to target them for screening may result in increased case detection and treatment coverage. But in contrast to high-income countries, screening for active TB and even LTBI in migrants in low- and middle-income countries may be more critical as incidence in migrants might be as high as that seen in poor inner-city populations [10, 26].

Another issue is that targeting migrants may have a discriminatory effect. Moreover, irregular migrants may not respond to these policies for fear of detection and criminal conviction or expulsion [27]. In order to overcome potential resistance to screening, free access to treatment and hospital admissions for TB cases among migrants must be guaranteed regardless of migration status [28].

### Tuberculosis diagnosis and treatment among migrants

TB-related Millennium Development Goals have brought substantial improvements in implementation of free diagnosis and TB treatment [1]. In Brazil, it was suggested that adding an intensive screening of household contacts of TB patients for active or latent TB infection to DOTS would reduce by 15% TB incidence in 5 years [25]. It further suggests that the strengthening of existing tools, as suggested in DOTS—though not fully implemented in several countries—might be effective in improving TB diagnosis and treatment in high-risk groups including migrants.

Although the proportion of cases of TB among migrants in middle-income countries is unknown, we estimate they account for a small proportion of cases. Few studies estimated the burden of TB among migrants in MIC, although migration has been increasingly important for disease control [26, 29–31], especially among the poorest and most vulnerable migrants. Institutional and skilled capacity to assist migrants need to be strengthened to deal with increasing migration flows from higher TB burden countries to large urban centres in MIC to ensure the provision of quality health care. However, the level of this demand is not yet clearly understood.

Free access to TB diagnosis and treatment has been implemented among migrants in many high-income countries, but access to health care can be especially difficult. Migrants face many social and cultural barriers to accessing treatment and sometimes they experience prejudice in health care settings [15]. Policies of free and universal access to health ensuring access for migrants has been a determinant for health equity promotion among South American migrants in large urban centres in Brazil [26]. Health care programs targeting specific groups in Latin American countries have contributed to improved access to health care [32], but the capacity of non-universal health systems to provide care to migrants may still be subjected to the current policy of a given country.

MDR-TB is currently a major challenge for TB control [1]. Migration from regions with high rates of MDR-TB is inevitable and potential issues must be addressed when planning control strategies. High coverage of drug susceptibility test (DST) among migrant populations must be achieved. MDR-TB rates are low in the Americas, which contrasts with high rates observed in sub-Saharan Africa and India [1]. Mathematical models have been increasingly built to understand TB transmission and health and economic impact of new tools and strategies for TB control including those for MDR-TB detection and treatment [33].

It is crucial for TB control to consider the characteristics of migrants in MIC, their interaction and social impact on host societies. Heterogeneity in TB incidence across different risk groups has been suggested as playing an important role in maintaining disease transmission, indicating that universal control measures are doomed to have limited impact if social inequalities are maintained [11, 34].

### Social protection policies

Conditional cash transfer programs have been successfully implemented in several high-, middle- and low-income countries in the past decade. Although inclusion criteria and benefits vary, these programs are usually integrated into education and health care policies. For example, the Brazilian conditional cash transfer (CCT) program “Bolsa Família” [35] provides families who have a per capita monthly income below USD 50 with a monthly stipend per child under 18. This is conditional on children attending school and family members seeking preventive care. This policy has had a positive impact on nutrition and food security of families, maternal and child mortality, utilization of health care services, and social determinants of some infectious diseases such as leprosy [35, 36]. The Mexican conditional cash transfer program “Oportunidades” has also had a positive impact on health outcomes and vaccination coverage among elders [37].

Few studies have focused on the impact of social protection policies on infectious disease burden, especially TB burden. Experimental provision of food baskets was associated to increased TB treatment completion and cure rates in Brazil [38]. There is also evidence showing a 7% higher cure rate among “Bolsa Família” beneficiares in Brazil [39], and a positive impact of social protection interventions (CRESIPT project) on catastrophic cost in poor households in Peru [40]. Siroka et al. [40] also suggested a strong negative correlation between expenditures as gross domestic product (GDP) percentage in social protection programs for poverty alleviation and TB rates (for incidence, prevalence and mortality) [41].

There is lack of data about social protection policies among migrants, but we suggest that poor migrants should be offered what is offered to poor locally born people. In Mercosul^4^, migrants have the same rights as nationals [42] and legal residents in Brazil can access CCT programs if poverty criteria are met, but irregular migrants, which could include person with free movement but under irregular labour conditions, continue to be poorer and neglected. Non-criminalization of migration is an essential effort to give migrants an opportunity to improve their economic status and consequently their health. To extend social protection benefits to migrants who meet delimited poverty criteria during TB treatment will improve migrant and community health. We suggest that social protection could be integrated into control programs and active TB screening among household contacts for example act as conditionalities. These actions must assure confidentiality to guarantee the absence of additional barriers to employment and protect migrants from being deported after treatment completion.

In middle-income countries with marked social inequalities such as Brazil, control strategies would be more successful if they take into account the intricacies of TB, placing control policies within the broader context of health care and social equity. Despite differences between public policies to promote social and health equity in Brazil and the circumstances in other middle-income countries, we believe that social protection policies must incorporate TB control strategies and a migration-related component. Migrants’ sociodemographic aspects, TB morbidity and mortality and migratory patterns specific to each country must be taken into account.

### The advantages of an intersectoral approach to control TB among migrants

The United Nations post-2015 agenda established more ambitious health targets and called for intersectoral policies. Since TB is a disease of poverty, predominantly affecting people living in large cities, and recognizing the wide heterogeneity existing between middle-income countries and source countries of migrants, from labour migrants to refugees, it is key to establish TB control programs alongside intersectoral public policies that promote social equity. Conditional cash transfer programs integrated into active TB screening and strong contact tracing could have a positive impact on TB control among the poorest and most vulnerable populations.

Cooperation and technology transfer among the BRICS and other low- and middle-income countries would also advance the control of neglected diseases [43]. These countries have reached agreements for cooperation and technology transfer for the control of neglected diseases, which points to greater integration and sharing innovation to the production of health inputs for TB control programs [43]. Some technical agreements between South American countries mediated by PAHO/WHO have given support to improve public primary health care programs in Paraguay and in the Andes regions [44]. Such strategies could be an example to other South-South cooperation initiatives.

These notable initiatives could be expanded to include more specific targets for TB control, as improving health care may not be sufficient to guarantee equity in access for migrants. Old and new TB control strategies may not have the desired impact unless there are initiatives for poverty reduction and early disease diagnosis among migrants. Moreover, because middle-income countries have limited health care resources, established health policies may be compromised if there is an increased demand due to migration and intersectoral policies are not planned.

## Conclusions

Middle-income countries and especially UMIC have been facing the same phenomena as seen in high-income countries in past decades: labour migration to large urban cities. Because of marked inequalities, big cities from middle-income countries must prepare to provide health care to incoming migrants, building on the reverse experience of being primarily a source of migration to more developed regions. In this context, intersectoral TB control strategies are needed to address social and health inequalities taking into account both migrants and locally born vulnerable groups in these countries. Conditional cash transfer policies may benefit poor migrants, especially during the first years after migration, and improve access to health care. Strengthening of DOTS and TB screening must also include migrants, especially irregular migrants that might not be supported by CCT programs. Finally, since migration flows largely take place within or between neighbouring regions [45], innovative control measures integrated with intersectoral and international policies are crucial for TB control and must be prioritized in regional agreements. Sending and receiving nations should channel their efforts to further integrating their health policies.

## Abbreviations

BRICS: Brazil, Russia, India, China, South Africa
CXR: Chest X-rays
DOT: Directly observed treatment, short-course
HIC: High income countries
HIV: Human immunodeficiency virus
IGRA: Interferon gamma release assay
LIC: Low income countries
LMIC: Low-middle income countries
LTBI: Latent tuberculosis infection
MDR-TB: Multidrug resistance tuberculosis
PLHIV: People living with HIV
TB: Tuberculosis
UMIC: Upper-middle income countries

## Footnotes

1 Although difficult to define, vulnerable groups include communities that are more vulnerable to conflicts, health conditions or economic shocks: people living in extreme poverty or socially excluded individuals, migrants or specific populations such as children, women and people with disabilities.

2 Russia is part of the BRICS group though it has been classified as high-income since 2013 and as upper-middle again in 2016.

3 Among a variety of definitions, “South” generally denotes low- to middle-income countries while “North” refers to high-income countries.

4 Mercosur full members include Argentina, Brazil, Bolivia, Paraguay, Uruguay and Venezuela. Associate members include Chile, Peru, Colombia, Ecuador, Guyana and Suriname (Mercosul, [46]).
